# Seminoma arising in splenogonadal fusion: a case report and literature review

**DOI:** 10.1186/s13000-023-01332-w

**Published:** 2023-03-30

**Authors:** Donglai Shen, Yuzhu Li, Yu Zhang, Xiao Chang, Xupeng Zhao, Jiabin Li, Xu Zhang, Gang Guo

**Affiliations:** 1 Department of Urology, The Third Medical Center of Chinese PLA General Hospital, 69 Yong Ding Rd, Beijing, 100039 China; 2 Department of Urology, State Key Laboratory of Kidney Diseases, Chinese PLA General Hospital/PLA Medical School, Beijing, 100853 China

**Keywords:** Splenogonadal fusion, Cryptorchidism, Testicular neoplasm, Congenital malformation

## Abstract

**Background:**

Splenogonadal fusion (SGF) is a rare congenital malformation in which the spleen is abnormally connected to the gonads or to the mesonephric derivatives. There is no obvious causality between SGF and testicular neoplasm. However, cryptorchidism, which is a well-known risk factor of testicular germ cell tumors, are the most frequent malformations associated with SGF. To our knowledge, there are only four reported cases of SGF associated with testicular neoplasm so far. Herein, we reported a patient of this condition, and briefly reviewed the related literature.

**Case presentation:**

A 48-year-old man was diagnosed with bilateral cryptorchidism 30 years prior, and only underwent a right orchiopexy for the left testicle could not be explored during the operation. At that time, doctors failed to realize the possibility of SGF due to the lack of sufficient knowledge of this condition. This time, the patient was treated for a left abdomen mass that was diagnosed as stage III metastatic seminoma. Then, a right orchiectomy, robot-assisted laparoscopic left retroperitoneal tumor resection, and left retroperitoneal lymph node dissection was performed after four cycles of BEP (bleomycin + etoposide + cisplatin) systemic chemotherapy in our center. The final diagnosis of SGF was made by postoperative pathology. The patient was re-examined in our center at 3 months and 6 months after the operation, and no obvious abnormalities were found.

**Conclusions:**

Surgeons should always bear in mind the possibility of association between bilateral cryptorchidism and splenogonadal fusion to avoid malignant transformation caused by delayed treatment.

## Background

Splenogonadal fusion (SGF) is a rare congenital malformation characterized by an abnormal connection between the splenic tissue and gonadal-mesonephric structures [1]. It was first described in 1883 by Bostroem [2]. In 1956, Putschar and Manion performed a detailed review of 30 SGF cases wherein the classification system of SGF was first proposed [3]. SGF lacks typical clinical symptoms and is usually associated with other congenital malformations such as inguinal hernia and cryptorchidism, or it may only involve testicular masses. Due to insufficient understanding of the disease, we often miss the best time for treatment or perform unnecessary orchiectomy because of a misdiagnosis [4–7].

In this article, we report a **patient** of SGF associated with seminoma in accordance with the CARE Guideline [8]. In fact, **patients** with SGF combined with **testicular germ cell tumor** are exceedingly rare, and to our knowledge, only four similar **patients** have been reported previously [9]. We identified all cases and reviews published in English by using the PubMed database and hope that our case and literature review will help to raise the clinicians’ awareness of this rare disease.

## Case presentation

**Fig. 1:**
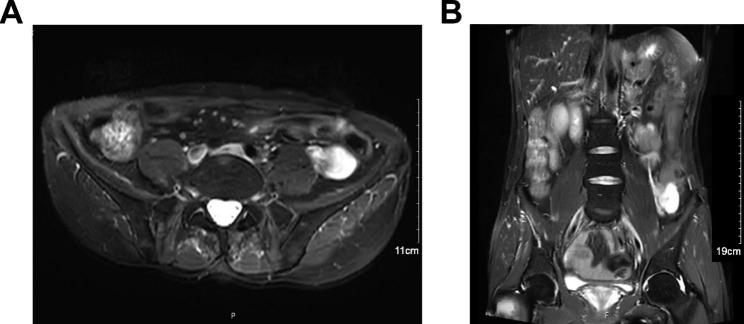
Preoperative abdominal enhanced MRI showed that the distal spleen and left retroperitoneal space occupying lesion (**6.7** cm in diameter), right inguinal space occupying lesion, left renal vein invasion, multiple retroperitoneal enlarged lymph nodes, and malignant tumor was considered. (A) cross section; (B) coronal section

**Fig. 2:**
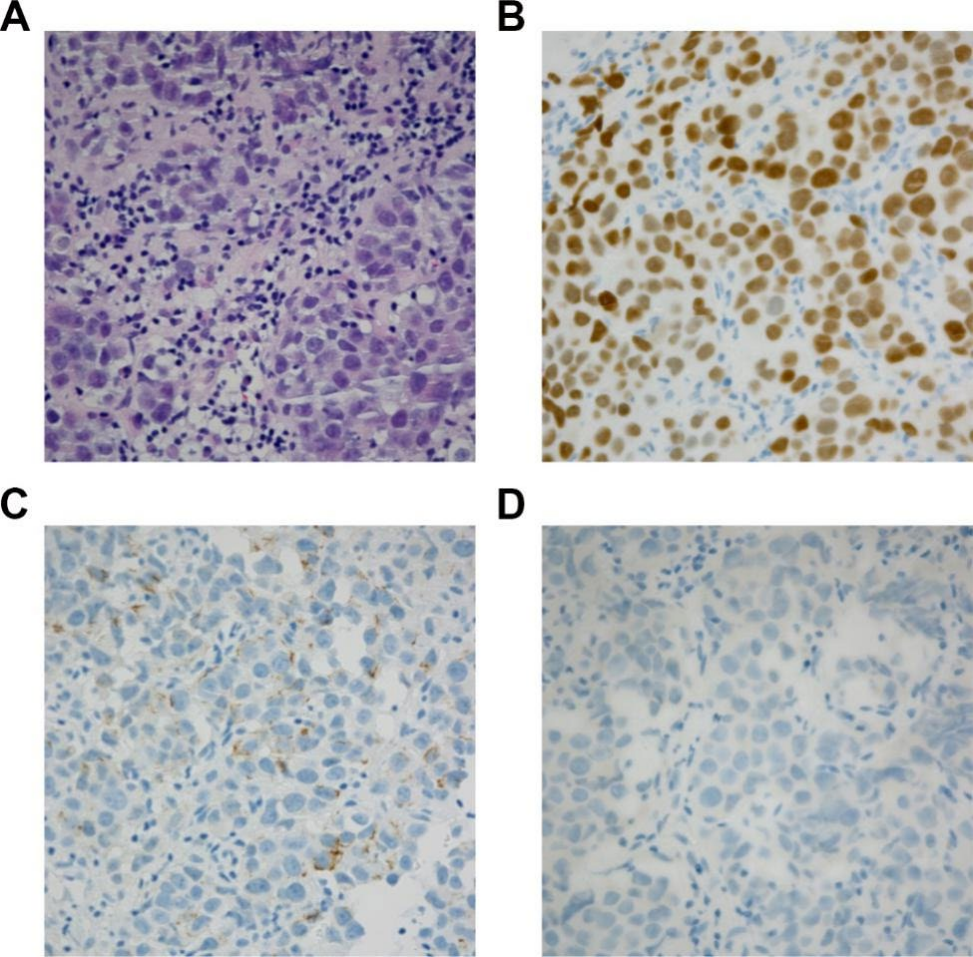
The pathological results of puncture biopsy of left retroperitoneal tumor before operation suggested that it was seminoma. (A) HE staining; (B) SALL-4 (+); (C) CK (-); (D) CD30 (-)

We report the case of a 48-year-old male who underwent right orchiopexy for bilateral cryptorchidism 30 years prior. However, the left testis could not be identified and no further examination was carried out. This time, the patient came to our hospital because he inadvertently found a painless mass in his left abdomen. Abdominal enhanced MRI (Fig. 1) suggested a distal splenic and left retroperitoneal space-occupying lesion (**6.7** cm in diameter) with left renal vein invasion, a right inguinal space-occupying lesion, and multiple retroperitoneal enlarged lymph nodes. Then, the patient underwent further needle biopsy of the left retroperitoneal tumor, and the pathological result combined with immunohistochemical staining (Fig. 2) suggested that it was a seminoma (CK (-), SALL-4 (+), PLAP (+), CD117(+), CD30 (-), GPC-3 (-), AFP (-), EMA (-), Vimentin (-), CD3 (-), CD20(-), LCA (-), ALK (-), Syn (-), CgA (-), S-100 (-), Ki-67 (40%)).

**Fig. 3:**
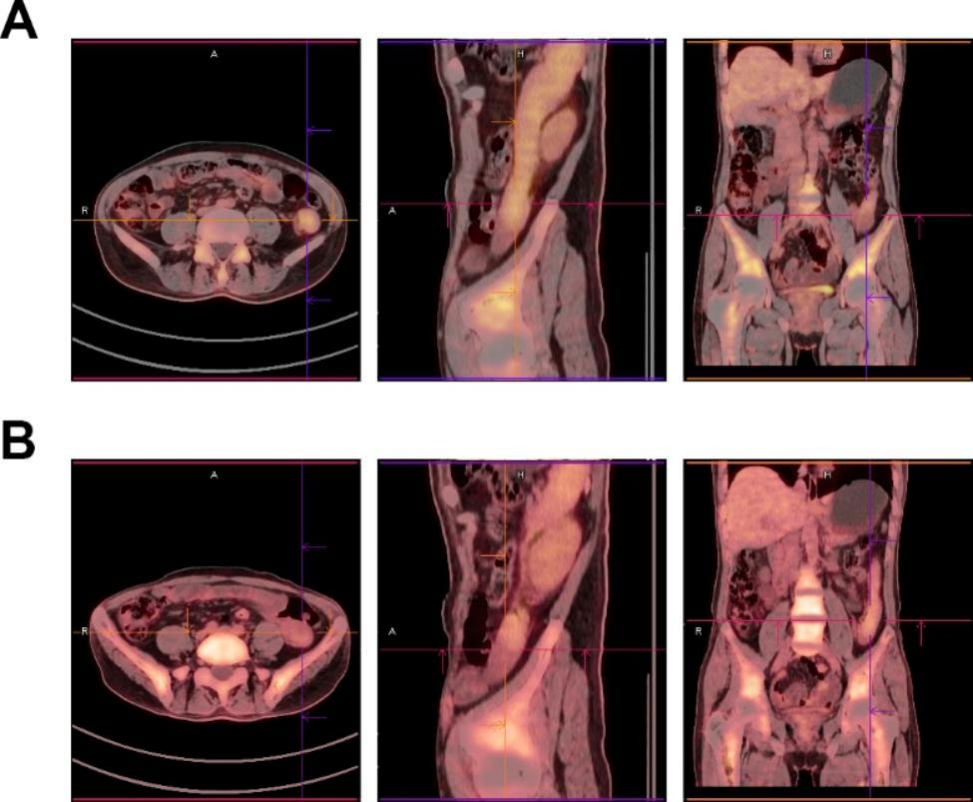
PET/CT after chemotherapy showed that the volume of both intra-abdominal tumor and retroperitoneal lymph nodes is smaller than before. (A) after two cycles; (B) after four cycles

Although serum tumor markers such as AFP, HCG and LDH were normal, combining the patient’s history, imaging examination and pathological examination, we considered the diagnosis of stage III metastatic seminoma. According to the guidelines [10], our center carried out four cycles of BEP (bleomycin + etoposide + cisplatin) systemic chemotherapy. No serious adverse reactions occurred during the course of treatment. The re-examination of PET/CT (Fig. 3A) after two courses of chemotherapy showed that hypermetabolic tumor could be seen in the inferior pole of the spleen, and its diameter (**5.1** cm) was smaller than before, hypermetabolic lymph nodes could be seen near the left renal vein, and their diameter was significantly smaller than before. After four courses of chemotherapy, PET/CT (Fig. 3B) showed that a slightly hypermetabolic tumor could be seen in the inferior pole of the spleen, and its diameter (**3.8** cm) was smaller than before. However, no abnormal metabolism was found in the left renal vein and retroperitoneal lymph nodes.

**Fig. 4:**
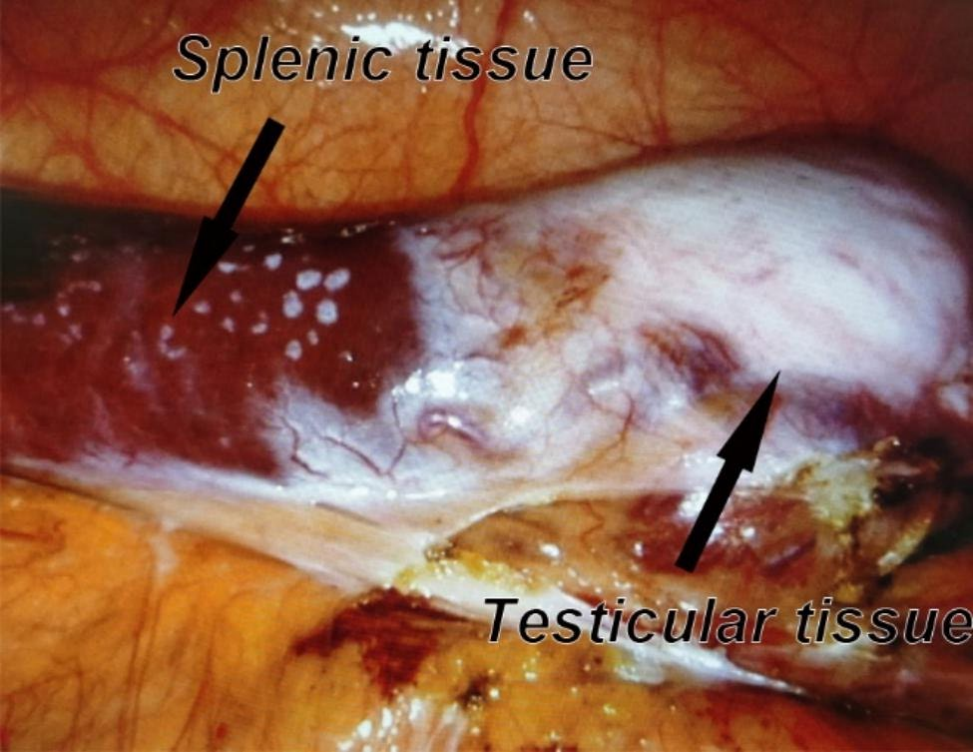
During the surgery, the spleen showed a long strip, and there was a pale mass fused with the inferior pole of it, which was considered as splenogonadal fusion

The PET/CT re-examination of the patient after four cycles of chemotherapy still showed that there was active residual tumor larger than 3 cm in diameter. Previous studies have suggested that the residual tumor can be surgically removed or re-evaluated by needle biopsy in this situation [11, 12]. However, the patient and his family refused to undergo another needle biopsy. Therefore, the patient underwent a robot-assisted laparoscopic left retroperitoneal tumor resection, and left retroperitoneal lymph node dissection in our center. **In addition, because the patient and his family were worried about the canceration of the right testis in the future and the preoperative examination showed that the right testis had no function, we performed right orchiectomy at the same time.** As shown in Fig. 4, during the operation, we observed that the shape of the spleen was abnormal, showing a long strip, and its inferior pole was lower than the inferior pole of the left kidney. In addition, we also observed a pale mass approximately 2.5 cm in diameter fusing with the inferior pole of the spleen, and testicular appendage-like vegetation could be seen below it. Through the combination of blunt and sharp separation, we carefully separated the tumor from the spleen and surrounding tissue, and removed it completely. In the process of separation, we also found that there were fiber bundles at the bottom of the mass, which were connected to the inner inguinal ring. The process of retroperitoneal lymph node dissection and right orchiectomy was not specific. Postoperative pathology confirmed that it was a mixture of splenic, testicular and epididymal tissue, and no clear tumor cells were found, which was consistent with the changes after chemotherapy. Therefore, we considered it to be splenogonadal fusion. **The patient was then treated with testosterone undecanoate, and the follow-up was carried out at 6 months and 1 year after operation. No obvious abnormality was found during the re-examination, and the patient was satisfied with the recovery of his sexual potency.**

## Discussion and conclusions

SGF is an extremely rare benign congenital malformation. It was reported for the first time in 1883 by Borstroem et al., and described in detail by Carragher in 1889 [2]. Until 1917, all the reported **patients** were found occasionally during autopsy studies. Therefore, only approximately 220 cases have been published in the literature [13]. In 1990, Carragher published a comprehensive review of 123 reported SGF cases [2]. Subsequently, Malik et al. reviewed 61 additional SGF cases in 2013 [14]. Since then, in 2021, Chen et al. reviewed 41 new SGF cases reported in English [13].

SGF is commonly seen in children and adolescents. The age of the patients is less than 10 years in half of the **patients** published, approximately 70% of the **lesions** occur in young men under the age of 20, and approximately 80% of the **lesions** occur in people under the age of 30 [15]. Due to the close proximity between the left gonad and spleen during embryological development, SGF mainly occurs on the left side (98% of **patients**) [16]. In addition, this condition is almost exclusively related to the male sex, with a male/female ratio of 14 ~ 15:1 [14, 17]. However, the reported male predominance is probably underestimated because **female gonads** are inside the body and thus are inaccessible for adequate clinical examination, and there are fewer complications related to **female gonads** than to **male gonads** [2, 6, 14]. Consistent with previous reports, the **lesion** in this report also occurred in a male, and the disease affected the left side. However, this patient was 48 years old, and the occurrence of this disease in this age group has been shown to be relatively rare in previous findings. In fact, the patient had gone to the hospital for bilateral cryptorchidism as early as when he was a teenager, but the doctor was not aware of the possibility of SGF at that time, which delayed the diagnosis and treatment.

The exact mechanism of SGF is not fully understood, but it is generally assumed that it occurs sometime during the 5th and 8th weeks of gestation, which is before the start of gonadal descent. Between the 5th and 6th weeks of gestation, the spleen develops from the splenic anlage in the left dorsal mesogastrium, and the gonadal ridge is formed between the mesonephros and dorsal mesentery. During the 6th and 7th weeks of gestation, the dorsal mesogastrium rotates to the left, placing the splenic anlage into close proximity with the left urogenital fold which contains the gonadal mesoderm. Such close proximity remains until descent of the gonads during the 8th week of gestation [1, 2, 14]. If abnormal fusion of the spleen and gonad occurs during this period, the splenic tissue attached to the gonad will descend along with the gonad. Therefore, the splenic tissue can appear anywhere in the gonadal descending path, even in the inguinal canal or scrotum.

In 1956, Putshcar and Manion classified SGF into two types [3]. The continuous type is characterized by the presence of a cord connecting the gonad and spleen, while in the discontinuous type, **ectopic** splenic tissue is directly fused to the gonad without connecting to the main spleen. Previous studies have shown that the incidence of the two types is equal [18], but other studies have revealed that the incidence of the continuous form is higher than that of the discontinuous form [9, 19].

Inguinal hernias and cryptorchidism are the most frequent malformations associated with SGF. Previous studies reported that approximately 30% of SGF patients had inguinal hernias or cryptorchidism [6], while approximately 60% of SGF **patients** with cryptorchidism were bilateral [20]. In addition, the frequency of congenital malformation in continuous SGF is 5 times higher than that in discontinuous SGF [19]. In continuous **SGF patients**, nearly 50% are accompanied by other congenital anomalies, the most common of which are micrognathia and limb anomalies. Other less common anomalies include cardiac malformations, cleft palate, hypospadias, spina bifida, varicocele, stenosis, and gastrointestinal malrotation [21–25]. The **patient** described in this manuscript is continuous SGF with bilateral cryptorchidism.

The clinical symptoms of SGF are not sufficiently specific, and clinicians do not have enough awareness of this rare disease. The diagnosis of SGF is usually made occasionally in pathological examination after surgery for cryptorchidism, inguinal hernia, or scrotal mass/swelling [1, 6, 14]. In particular, the discontinuous type of SGF often presents as a painless scrotal mass without other malformations, imitating a testicular tumor. Unnecessary orchiectomy was performed in up to 37% of these patients [2]. Unless the patient suffers from a disease involving the spleen, such as salmonellosis, malaria, mononucleosis, and leukemia, ectopic splenic tissue will also be affected, resulting in symptoms of acute testicular swelling and pain [20].

To the best of our knowledge, this is the fifth reported case of **testicular germ cell tumor** arising in SGF thus far [9, 26–28]. In fact, there is no obvious causality between SGF and malignant transformation, and we believe that this condition is mainly related to cryptorchidism. The most common sites of cryptorchidic testicles are the inguinal canal (63%), external inguinal ring (9%) and abdomen (2%), of which 80% are palpable, while 20% are unpalpable [29]. Cryptorchidism is a well-known risk factor of malignant testicular tumors. If cryptorchidism is not treated by orchidopexy at an early stage of life, the probability of malignant transformation of a cryptorchid testicle is much higher than that of a normal testis, and the most common pathological type is seminoma. Moreover, the location of cryptorchidism also affects the risk of testicular malignant transformation. The higher the location of the cryptorchid testicle is, the higher the risk of malignant transformation, and approximately half of abdominal cryptorchidism **patients** finally develop into **testicular germ cell tumor** [29]. Therefore, the European Association of Urology (EAU) guidelines for pediatric urology suggests that “If a testis has not concluded its descent by the age of six months, surgery should be performed within the subsequent year, and by age 18 months at the latest” [30]. In fact, this patient had been treated in the hospital for bilateral cryptorchidism when he was as young as 18 years old, but due to the lack of sufficient knowledge of SGF, doctors only made a diagnosis of cryptorchidism and failed to realize the possibility of this condition. At that time, this patient only underwent right orchiopexy because the left testicle could not be explored during the operation, so the best time for treatment was delayed, which increased the risk of malignant transformation of the left ectopic testicle. This time, the patient came to our center for a left abdominal mass and was diagnosed with stage III metastatic seminoma, which was finally confirmed as SGF by postoperative pathological examination. We suspect that this may be due to the pathological complete remission caused by the four courses of systemic chemotherapy before surgery, so that, no definite tumor cells were found in the postoperative histological examination of this patient. However, PET/CT still indicated that the residual tumor was active in this **patient**, which may be due to the false-positive rate of this examination [12]. In addition, we further compared the current case with the previously reported 4 cases of **testicular neoplasm** associated with SGF. As shown in Tables 1, all 5 patients were over 20 years old, including 2 over 30 years old and 1 over 40 years old. In all 5 **patients**, the left side was affected, of which 4 **patients** were continuous SGF with cryptorchidism, and malignant transformation occurred in intra-abdominal testicles, while 1 **patient** was a scrotal mass associated with discontinuous SGF, and canceration occurred after orchiopexy. In terms of tumor markers, the levels of HCG and/or AFP were abnormally elevated in 3 patients, while those in the other 2 patients were not elevated. In terms of histological types, 3 **specimens** were seminoma, 1 was **mixed malignant germ cell tumor** and 1 was **non-seminomatous germ cell tumor**.

**Table 1:** Comparison of present and previous cases of testicular malignancy arising from splenogonadal fusion (SGF).

Source, y	Age, y	Clinical and laboratory features	Preoperative diagnosis	Final histological diagnosis	Type	Treatment	Follow-up
Falkowski and Carter, 1980 [26]	38	Bilateral cryptorchidism, intra-abdominal tumor, and elevated HCG in serum	Bilateral cryptorchidism with intra-abdominal tumor, ectopic spleen	SGF with anaplastic seminoma	Continuous	Excision of testicular tumor and spleen with radiation therapy to abdomen, mediastinum, and both supraclavicular regions	Not reported
Thomsen et al., 1997 [27]	28	Status post left orchiopexy for left cryptorchidism, left testicular mass, and elevated serum AFP	Testicular tumor	SGF with mixed germ cell tumor (endodermal sinus tumor, immature teratoma, and seminoma in situ)	Discontinuous	Left orchiectomy and excision of attached ectopic splenic tissue	No evidence of disease 9 months after surgery
Imperial and Sidhu, 2002 [29]	27	Bilateral cryptorchidism, intra-abdominal tumor, and elevated HCG and AFP in serum	Bilateral cryptorchidism with intra-abdominal testicular cancer	SGF with non-seminomatous germ cell tumor (embryonal carcinoma and yolk sac tumor)	Continuous	Excision of left testicular tumor and spleen, right orchiopexy, and 3 cycles of BEP (cisplatin, etoposide, and bleomycin) chemotherapy	No evidence of disease 5.5 years after surgery
Lopes et al., 2012 [9]	36	Bilateral cryptorchidism, intra-abdominal tumor, and normal tumor markers	Bilateral cryptorchidism with infertility, SGF	SGF with seminoma in situ	Continuous	Excision of left testicular tumor, right orchiopexy, and left testicular prosthesis implantation	Not reported
Present case	48	Bilateral cryptorchidism, intra-abdominal tumor, and normal tumor markers	Bilateral cryptorchidism with stage III metastatic seminoma	SGF with seminoma	Continuous	4 cycles of neoadjuvant chemotherapy (BEP), excision of testicular tumor and small amount of attached spleen, and dissection of retroperitoneal lymph node	No evidence of disease 6 months after surgery

In recent years, with better awareness of this disease and the improvement of examination techniques, the diagnosis of SGF has been more accurate accordingly [13]. When we encounter an abnormal gonad, such as testicular mass or cryptorchidism, SGF should be considered, and ultrasound is the first choice for preliminary screening. For continuous SGF, the cord between the left testicle and spleen can be visualized by ultrasound, while for discontinuous SGF, the ectopic splenic tissue is usually visualized as a well-encapsulated, extra-testicular homogenous hypoechoic or isoechoic mass [31, 32]. However, the diagnostic accuracy of ultrasound is affected by the experience of the examiners. Meanwhile, CT and MRI are reliable and accurate in detecting the position and shape of the testis and for ruling out other congenital malformations [33]. Moreover, Technicium-99 m liver-spleen scan (99mTc) could detect an accessory spleen and thus could be employed for diagnosis when a surgeon has a high suspicion of SGF before surgery [6]. If the above methods are unsuccessful, an intraoperative biopsy should be applied to confirm the nature of the mass [6]. On this basis, Youssef et al. [34] proposed a diagnosis and treatment decision tree for SGF: ultrasound and tumor marker testing should be employed first. If tumor markers are elevated, orchiectomy can be performed directly; if tumor markers are normal but a testicular neoplasm is highly suspected by ultrasound, surgical exploration and intraoperative biopsy will be feasible for diagnosis. If tumor markers are not elevated and if it is difficult to make a diagnosis on ultrasound, 99mTc can be further applied. If there is radioactive tracer fixation in the scrotum and the spleen, resection of the ectopic splenic tissue should be performed, and if there is no radioactive tracer fixation, surgical exploration and intraoperative biopsy should be used. In this case report, the patient initially went to the hospital for bilateral cryptorchidism without any diagnoses of other congenital malformations. Due to the lack of advanced examination methods and the in-sufficient understanding of the disease 30 years ago, the diagnosis of SGF was missed. However, the left testicle was not detected during the surgery at that time, suggesting that if such gonadal abnormalities are encountered in our future clinical work, especially those involving the left side, the possibility of SGF should be considered and further examination should be carried out to confirm the diagnosis to avoid a delay in treatment.

In conclusion, SGF is a rare congenital benign disease that is often diagnosed incidentally during treatment for cryptorchidism, scrotal mass or inguinal hernia in young patients. It may not increase the risk of malignant transformation alone. However, cryptorchidism, as its accompanying symptom, increases the risk of **testicular germ cell tumor**. Surgeons, especially pediatric surgeons, should always bear in mind the possibility of this condition and use imaging examination, 99mTc and surgical exploration **with** extemporaneous examination to improve the diagnostic accuracy of this disease. Only in this way can we avoid increasing the risk of malignant transformation due to delayed treatment or affecting the normal life and mental health of patients due to unnecessary orchiectomy.

## Data Availability

Not applicable.

## Abbreviations

SGF: Splenogonadal fusion
BEP: bleomycin + etoposide + cisplatin
EAU: the European Association of Urology
99mTc: Technicium-99 m liver-spleen scan
MRI: Magnetic resonance imaging
PET/CT: Positron emission computed tomography
HCG: Human chorionic gonadotropin
AFP: alpha-fetoprotein
CK: Cytokeratin
SALL: Spalt like transcription factor
PLAP: Placental alkaline phosphatase
CD: Cluster of differentiation
GPC: Glypican
EMA: Epithelial membrane antigen
LCA: Leukocyte common antigen
ALK: Anaplastic lymphoma kinase
Syn: Synaptophysin
CgA: Chromogranin A
